# The Roles of H_2_O/Tetrahydrofuran System in Lignocellulose Valorization

**DOI:** 10.3389/fchem.2020.00070

**Published:** 2020-02-07

**Authors:** Jianmei Li, Wenyu Zhang, Shuguang Xu, Changwei Hu

**Affiliations:** Key Laboratory of Green Chemistry and Technology, Ministry of Education, College of Chemistry, Sichuan University, Chengdu, China

**Keywords:** water, tetrahydrofuran, co-solvent, lignin, hemicellulose, cellulose, value-added chemicals

## Abstract

Lignocellulosic biomass as a potential alternative to fossil resource for the production of valuable chemicals and fuels has attracted substantial attention, while reducing the recalcitrance of lignocellulosic biomass is still challenging due to the complex and cross-linking structure of biomass. Solvent system plays important roles in the pretreatment of lignocellulose, enabling the transformation of solid biomass to liquid fluid with better mass and heat transfer, as well as in the selective formation of target products. In particular, H_2_O/tetrahydrofuran (H_2_O/THF) system has recently been widely applied in lignocellulose valorization, which has been proved to exhibit outstanding efficiency for the conversion of lignocellulose, solubilization of the intermediates and products, and shifting reaction equilibrium, thereby significantly improving the yield and selectivity of target products, as well as the full utilization of lignocellulose. In addition, THF shows low toxicity, and is known as a renewable solvent which can be produced from bio-derived chemicals. Herein, this review concentrates on the advances of H_2_O/THF system in lignocellulose valorization in recent years. Several aspects relative to the roles of H_2_O/THF system are discussed as follows: the pretreatment of lignin, conversion of hemicellulose and cellulose components in lignocelluloses, and the promoting formation of valuable chemicals like furfural, 5-hydroxymethyl furfural (HMF), levulinic acid, and so on, as well as the inhibiting role in humins formation. This review might provide useful information for the design of effective solvent system in full utilization of lignocellulosic biomass.

## Introduction

**Figure 1:**
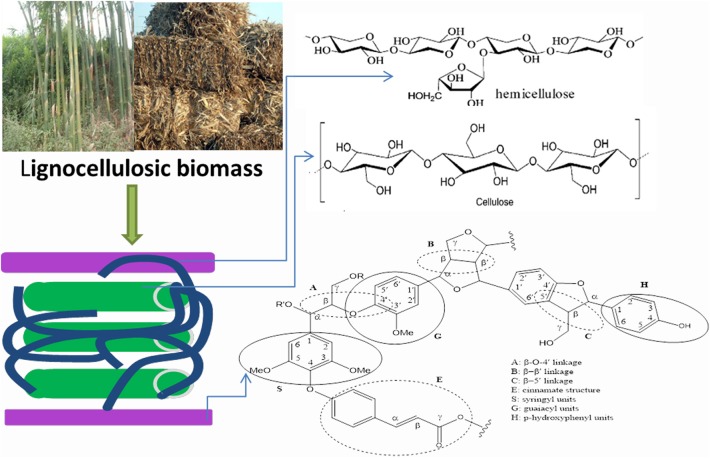
The structure of lignocellulosic biomass.

The over-exploitation of fossil resources and the resultant environmental concerns have impelled the development of renewable alternative feedstock to replace the depleting fossil resources. In recent years, increasing interests have been focused on the valorization of renewable lignocellulosic biomass instead of the conventional fossil resources for the production of value-added chemicals and biofuels (Lim et al., 2012; Lin et al., 2013). Every year, about 220 billion tons of dry biomass (ca. 45 EJ of energy content) can be obtained, and lignocellulosic biomass occupies about 70–95% of it. Cellulose, as the major component (40–50%) and elementary fibrils of lignocellulosic biomass, is a homopolysaccharide, comprised of D-glucose units via β-1,4-glucosidic bonds (Figure 1). It has high degree of crystallinity, polymerization (from 100 to 20,000) with high molecular weight, caused by the abundant hydrogen bonds between different anhydroglucan chains. Hemicellulose, surrounding the cellulose fibrils, bonds with cellulose and forms the gel matrix. Hemicelluloses (occupied 25–35% of the lignocellulose) are amorphous polysaccharides, and are made up of mainly two pentoses (xylose and arabinose) and three hexoses (galactose, glucose, and mannose). Compared to cellulose, hemicellulose has lower degree of polymerization (80–200). Different from carbohydrate-based cellulose and hemicellulose, lignin (occupied 15–20% of the lignocellulose) with high molecular weights in a range of 600–15,000 kDa, is comprised of three phenyl-propanols linking with each other via C-O linkages (e.g., β-O-4,α-O-4, and so on) and C-C linkages (e.g., β-β′, β-5, and 5–5′ linkages, etc.) (Zhou et al., 2011; Tuck et al., 2012). Via effective utilization of the natural structure of raw lignocellulose, various platform chemicals and fine chemicals can be produced. Typically, xylose, furfural, and acetic acid can be produced from hemicellulose in lignocellulose. Glucose, fructose, sorbitol, ethylene glycol, 5-hydroxymethyl furfural (HMF), levulinic acid (LA) as well as lactic acid, can be obtained from the conversion of cellulose component in lignocellulose (Kunkes et al., 2008). Among these platform chemicals, furfural, HMF, and levulinic acid have been recently defined as the top candidates of future bio-based chemicals (Zakzeski et al., 2010; Cai et al., 2014a). From the degradation of lignin in lignocellulosic biomass, many aromatic compounds, such as guaiacol, 4-ethylphenol, 4-vinylphenol, 2,6-dimethoxylphenol, and so on, can be obtained (Collard and Blin, 2014; Graglia et al., 2015). Most of these aromatic compounds can be used as the feedstock for the synthesis of polymers and materials with novel interesting properties. However, the complex composition and cross-linked structure of these three main components make the degradation of lignocellulose challenging.

Liquefaction in the presence of solvent has been considered as an efficient approach for the production of valuable chemicals or biofuels from lignocellulose, since solvents significantly improve the mass and heat transfer of solid lignocellulosic biomass (Li et al., 2018). Compared to pyrolysis, solvent liquefaction requires relatively moderate reaction temperature. Importantly, it also greatly improves the reaction rates, selectivity and the stability of desired products, as well as the economics of downstream separation. Moreover, solvent liquefaction (usually in H_2_O solvent) endures the presence of water in lignocellulose feedstock, thus eliminating the process of drying raw feedstock. Several studies have suggested that the mixture of polar aprotic solvent with water in lignocellulose conversion can promote the solubility of substrate fractions and influence the chemical reaction thermodynamics, thus leading to high reaction rates and improving the selectivity toward target products, where either homogeneous catalysts (acids, alkaline) or heterogeneous catalysts are usually employed (Liu et al., 2015; McCallum et al., 2018; Chen et al., 2019; Maneechakr and Karnjanakom, 2019). The type and properties of solvent not only determines the distribution of products and their yields, but also affects the subsequent separation of target products (Pace et al., 2012; Shuai et al., 2016). Shuai and Luterbacher (2016) divided the solvent effects into two categories: effects on the solubility of biomass components and their derivatives, and effects on chemical thermodynamics, which could be represented by considering the typical kinetic expression of a first-order reaction rate: *r* = *k* × *C*. Here, *r* represents the reaction rate and *k* represents the rate constant. *C* is the reactant concentration. Due to the low concentrations (*C*) of insoluble biomass in solvent, the reaction rates of biomass conversion are often restricted. Therefore, enhancing the biomass solubility can effectively improve the reaction rate of biomass conversion. Besides, increasing the rate constant (*k*) can also promote biomass conversion, which can be achieved by using a solvent that affects the thermodynamics of the molecules and complexes that participate in the chemical reaction.

Recently, H_2_O/tetrahydrofuran (H_2_O/THF) mixture has been proved to act as a promising solvent system with multi-functions for the fractionation of lignocellulose and solubilizing the resulted derivatives to obtain valuable chemicals with high yield and selectivity. In addition, THF can be synthesized from biomass-based derivatives, such as furfural, maleic anhydride, or 1,4-butanediol, thus is a promising renewable and green solvent. Table 1 summarizes the advantages and disadvantages of THF system compared to other solvents for the conversion of biomass. Herein, this review tends to concentrate on the advances and roles of H_2_O/THF mixture in lignocellulose valorization in recent years.

**Table 1:** The comparison of THF with other solvents.

**Organic solvents**	**Properties**			**Advantages, disadvantages for biomass conversion**
	**Density (g cm^−3^)**	**Boiling point (K)**	**Solubility in water**	
DMSO	1.1	462.1	Miscible	Poor solubility, environmental unfriendly and non-renewable, difficult recycle, high boiling point
Dioxane	1.0	374.0	Miscible	Poor solubility, environmental unfriendly and non-renewable
Water	1.0	373.1	–	Green, safe, abundant, but limited solubility for lignin
THF	0.9	339.0	Miscible	Easy separation and recycle, renewable, but poor solubility for cellulose and hemicellulose
Ethanol	0.79	351.3	Miscible	Safe, renewable, easy separation and recycle, but limited solubility for biomass
GVL	1.05	480.1	Miscible	Safe, renewable, good solubility for lignin, but high boiling point leads to difficult separation and recycle

## The Properties of H_2_O/THF Co-solvent

THF has been employed as a solvent and intermediate in industry for decades. THF has low boiling point (66°C) with a high vapor pressure (21.6 kPa at 25°C), which enables it easy recovery. In general, it is known that THF is highly polar and miscible with water, forming an azeotrope with water (Howard, 1990; Kroschwitz et al., 2004). Liu et al. (2019) performed *ab initio* molecular dynamics simulations, and proved that hydrogen bond was formed between the H atom of H_2_O and O atom of THF with a 1.694 Å of bond length. However, Matouš et al. (1972) demonstrated that H_2_O/THF co-solvent had great positive deviations from Raoult's law to such degree. H_2_O/THF co-solvent passed through a temperature regime where THF and H_2_O became immiscible, in which a transition from a transparent mixture to opaque medium could be observed. That is to say, H_2_O/THF co-solvent exhibits an unusual closed-loop miscibility gap, in which H_2_O and THF are mixed when the temperature is both below a certain temperature point and above a second value, while they spontaneously demix between this temperature gap. The region of limited miscibility is close and bounded by the mass fraction ω_*THF*_ ϵ (0.28, 0.72) and by temperature *t* ϵ (71.8, 137.1)°C (Fowles et al., 2013).

It is also considered that the presence of THF in H_2_O/THF co-solvent has potential harm to both human and animal health. To investigate the toxicity of THF, Fowles et al. (2013) presented a detailed review to discuss the toxicological, exposures, risks and environmental hazards of THF. It is indicated that THF is neither a sensitizer nor irritant for skin. THF is non-mutagenic according to the studies both *in vitro* and *in vivo*. In addition, the low log*K*_ow_ value (<3) implies that THF has low bioaccumulation. THF is not persistent in environment as it can be biodegraded. Therefore, it is considered that THF shows low acute toxicity, and it is not necessary to pose a concern on its hazards to both human and environment, as it is utilized and managed in current use (Cheng et al., 2007; Chen et al., 2013).

## Lignin Solubilization and Depolymerization in H_2_O/THF System

The extensive covalent cross-linking of lignin with hemicellulose (or cellulose) in lignocelluloses limits the accessibility to carbohydrates and prevents the extraction of polysaccharides. Therefore, lignin removal is usually necessary in many industries, such as paper industry and bio-ethanol production, in which lignin is firstly fractionated and conversion from lignocellulose as a “lignin-first” approach. However, the efficient solubilization of lignin is still challenging. It is reported that the solubility of lignin in solvent is related to the Hildebrand solubility parameter (δ), which is defined as follows (Schuerch, 1952; Ni and Hu, 1995; Meng et al., 2018; Wang et al., 2018).

$$\begin{array}{l} \delta \;=\;{\left(E/V\right)}^{1/2} \end{array}$$

Here *V* represents the molar volume of solvent. *E* represents the vaporization energy at free pressure, which is constituted by three parameters corresponding to hydrogen bonding forces, non-polar/dispersion forces, and dipole forces, showing as follows:

$$\begin{array}{l} \delta^{2}\;=\;\delta_{\text{D}}^{2}+\delta_{\text{P}}^{2}+\delta_{\text{H}}^{2} \end{array}$$

Here, δ_D_ represents the dispersion interaction and δ_P_ is polar interaction, while δ_H_ represents the hydrogen bonding interaction. It is considered that solvent, whose δ-values is approximate to 22.5 MPa^1/2^, shows good solubility for lignin. The δ value of water is 23.4 (cal/cm^3^)^1/2^, and the δ value of THF is 9.5 (cal/cm^3^)^1/2^. H_2_O/THF co-solvent is commonly considered as a good solvent for lignin (Mlynar and Sarkanen, 1996; Hansen, 2007). For example, Xin et al. (2012) used HSP to study the characteristics of several solute–solvent pairs, including three organic solvents (1,4-dioxane, ethyl acetate, and THF) and 1-ethyl-3-methylimidazolium acetate, and proved that THF was the best solvent for lignin among the three investigated solvent systems, followed by dioxane and ethyl acetate. In the process of lignin dissolution in H_2_O/THF co-solvent, it is considered that H_2_O molecules with smaller molecular size than organic solvent can act as an efficient plasticizer, which favors organic solvent diffusion into the compact lignin complexes, thereby leading to the solubilization of lignin in water-organic solvent mixture (Wang et al., 2018; Meng et al., 2019).

Recently, a “lignin-first” strategy, called as Co-solvent Enhanced Lignocellulosic Fractionation (CELF), has been widely applied in the fractionation of lignin from raw lignocellulose with high efficiency, in which H_2_O/THF co-solvent is employed as the solvent system with the help of dilute acid (Nguyen et al., 2015; Smith et al., 2016a,b,c). In H_2_O/THF mixture, about 85–90% of lignin in raw lignocellulosic biomass can be fractionated and solubilized. After boiling off the THF solvent, a lignin product without ash and sugar can be obtained by precipitation, which can act as a potential feedstock and be upgraded to high value-added chemicals and fuels in the further bio-refinery (Zhuo et al., 2018). The pretreatment in H_2_O/THF system drastically reduces the molecular weight of lignin, and the cross-condensation reactions can be also efficiently minimized. For example, Meng et al. (2018) investigated the CELF lignin structure isolated under different conditions, and revealed that the molecular weight of lignin obtained sharply reduced by up to ~90% when compared to the native lignin. Moreover, the extensive cleavage of β-O-4 linkages in lignin after pretreatment was also found, in addition to an obvious decrease of aliphatic OH groups resulting from the oxidation of side chains in lignin. However, the amount of phenolic OH groups greatly increased caused by the breakage of inter-unit linkages in lignin (Cai et al., 2013, 2014a,b; Nguyen et al., 2015).

**Figure 2:**
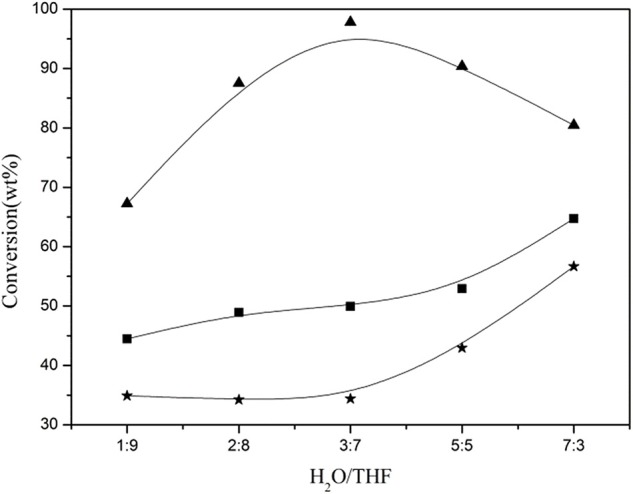
Effect of the ratio of H_2_O/THF on the conversion of corncob residue (■ raw material, ⋆ cellulose converted/cellulose contained, ▴ lignin converted/lignin contained; reaction conditions: corncob residue: 4.0 g, reaction temperature: 220°C, reaction time: 2.0 h). Reproduced from Jiang et al. (2014) with permission from Royal Society of Chemistry.

Zhang et al. (2018) compared the performances of several solvents, including THF, 2-methyltetrahydrofuran (MeTHF), γ-valerolactone (GVL), ethyl acetate (EAC), and γ-butyrolactone (GBL), on the solubilization of lignin in corncob residue, and indicated that miscible co-solvents (e.g., H_2_O-THF, H_2_O-GVL, and H_2_O-GBL) generally showed better ability for lignin fractionation than both single solvents and immiscible co-solvents like H_2_O–MeTHF and H_2_O–EAC under mild temperature. It was revealed that H_2_O and organic solvent showed significant synergetic effect which favored the cleavage of linkages between lignin and amorphous cellulose in corncob residue. Organic solvent with different structure and property also influenced the solubilization of lignin with various structure units. For instance, MeTHF, EAC, and GVL preferentially dissolved S- and G-type lignin, while THF solvent showed high selectivity to fractionate and solubilize lignin with G and H units. In H_2_O–THF, H_2_O–MeTHF, H_2_O–EAC, and H_2_O–GBL co-solvents, β-γ bond was easier to be broken than β-O-4 bond, thereby giving 4-ethylguaiacol and 4-ethylphenol at higher temperature which occupied ~70% of the total determined monophenols. In contrast, in H_2_O–GVL co-solvent, α-1 bond was firstly broken, yielding guaiacol as the main product which occupied ~75% of the total determined monophenols. Jiang et al. (2014) investigated the conversion of lignin in corncob residue in H_2_O/THF co-solvent, and revealed that H_2_O and THF solvents showed a synergistic effect on facilitating the dissolution of lignin, in which the ratio of H_2_O to THF played an important role. It was found that the conversion of lignin in corncob residue initially increased with an increasing ratio of H_2_O/THF, and got a maximum (89.8%) at the ratio of 3:7 (v/v), then decreased (Figure 2). H_2_O, as a nucleophile agent, was necessary to break the linkages between lignin and cellulose components, such as hydrogen bonds, ether and ester bonds, ascribing to its high hydrogen bond acceptor ability. Due to the lower hydrogen bond acceptor ability of THF than that of H_2_O, THF mainly presented a core effect on dissolving the fragment of dissolved lignin. The fractionated and solubilized lignin existed as oligomers with little monophenolic compound formation. When the solubilized lignin was further converted at higher temperature (300°C) in single THF solvent, the yield of monophenols was significantly enhanced. In particular, the yield of 2,6-dimethoxylphenol, 4-ethylguaiacol and 4-ethylphenol sharply increased to 6.6, 4.0, 10.5 wt%, respectively. The yield of determined monophenols was raised to 24.3 wt% in the absence of extra hydrogen source addition. It was considered that the special properties of THF as a supercritical fluid under the reaction conditions contributed to the great increase of monophenol yield at 300°C. Thus, they proposed that THF solvent significantly favored the depolymerization of resulted lignin oligomers yielding monophenols under severe reaction conditions. Meng also investigated the solubilization of CELF lignin in several solvents. Results showed that 50 mL H_2_O/THF co-solvent (40% THF) could solubilize about 2 g of lignin. 30% THF/H_2_O co-solvent gave the maximum fraction yield (~51%), however, there was no obvious relationship between the THF content and the fraction yield (Meng et al., 2019). Both molecular weights of the individual fractions obtained sharply decreased with the decrease of THF concentration from 35 to 20%.

The introduction of catalyst, especially alkaline, into H_2_O/THF system could selectively improve the solubility and depolymerization of fractionated lignin (Nakasaka et al., 2017). For instance, it was pointed out that, with the introduction of MgO, much higher phenolic monomer yield (13.2%) was obtained in THF solvent than that without catalyst, attributing to the excellent fractionation and dissolution of lignin and the promoting effect on the catalytic activity of MgO in THF (Table 2; Long et al., 2014). Furthermore, it was considered that O atom of THF could coordinate with Lewis acids, thereby increasing the basicity of catalyst. When Na_2_CO_3_ was introduced into H_2_O/THF co-solvent, it was found that almost all hydrogen bonds, ester and ether bonds between the cellulose and lignin components in corncob residue were broken at 140°C, leading to 94.6% removal of lignin (Jiang et al., 2016). In addition, the Cα-Cβ in aliphatic side-chain of lignin as well as β-O-4 linkage could be cleaved, yielding aryl aldehydes. In this work, H_2_O was considered to contribute to the breakage of weak inter-linkages between lignin and cellulose components in corncob residue, and THF primarily solubilized the resulted fragments under mild temperature. Higher temperature (300°C) enabled the further cleavage of C_Ar_-Cα bond, selectively giving monophenols with limited substituted alkyl groups. In the absence of extra hydrogen sources, 26.9 wt% yield of monophenol was achieved.

**Table 2:** The effect of reaction medium on lignin conversion.

**Solvent**	**Catalyst**	**Conversion of lignin (%)**	**Yield of phenolic monomer (%)**				**References**
			**Phenol**	**Guaiacol**	**Syringol**	**Others**	
CH_3_OH^a^	MgO	90.7	0.25	1.36	1.19	6.46	Long et al., 2014
C_2_H_5_OH^a^	MgO	84.5	0.25	1.42	1.24	5.54	Long et al., 2014
C_2_H_5_OH/H_2_O^a^	MgO	92.5	0.82	1.58	1.32	7.52	Long et al., 2014
THF^a^	MgO	97.5	0.75	2.81	1.45	7.79	Long et al., 2014
H_2_O^a^	MgO	42.3	0.23	0.70	0.52	0.74	Long et al., 2014
H_2_O/THF^b^	Na_2_CO_3_	–	7.4	5.2	6.0	4.8	Jiang et al., 2016
H_2_O/THF^c^	Na_2_CO_3_	–	5.6	4.9	12.2	4.2	Jiang et al., 2016
THF	–	–	6.5	2.79	1.01	1.39	Jiang et al., 2014

a *Condition: solvent 40 mL, MgO 3.0 mmol, and lignin 0.5 g, 250°C, 30 min*.

b *In step-one: Na_2_CO_3_ was introduced into 100 mL solvent (H_2_O/THF: 3:7, v/v), 140°C*.

c *In step-one: Na_2_CO_3_ was introduced to 100 mL solvent (H_2_O/THF: 5:5, v/v), 140°C*.

Given the outstanding lignin solubility in H_2_O/THF co-solvent, many efforts have been devoted to reveal the solubilization mechanism of lignin in H_2_O/THF co-solvent. Smith et al. (2016a,b) used temperature replica-exchange molecular dynamics to simulate lignin structure in H_2_O/THF co-solvent, and revealed that single water media was a bad solvent for lignin, in which polymer–polymer interactions were favored. In this case, the lignin polymer collapsed to “globular” conformation, in which monomers were tightly packed. On the contrary, H_2_O/THF co-solvent acted as a “theta” solvent, wherein lignin adopted a random coil state, unlike the collapsed globular conformation in single water medium. Because the interactions between polymer and solvent was preferential to the interactions between polymer and polymer, the self-aggregate of lignin with coil conformation could not happen, thereby resulting in the easy removal of lignin. Since H_2_O/THF co-solvent system passed through a temperature gap in which H_2_O and THF became immiscible, they also investigated the conformation of lignin in H_2_O/THF co-solvent at this temperature regime. At the ratio of H_2_O to THF commonly adopted in the process of lignin pretreatment, H_2_O and THF became immiscible near 348 K and turned to miscible again above 410 K, which was just lower than the temperature (445–475 K) of lignin pretreatment in CELF approach (Smith et al., 2016b). It was worthy of noting that lignin was still a flexible random coil in H_2_O/THF co-solvent for *T* ≥ 303 K (Smith et al., 2018). At lower temperature (*T* = 283 K), it was found that lignin did swell in H_2_O/THF co-solvent when compared to that in single water, although it did not exist in a random-coil conformation. This indicated that the presence of THF promoted the transform of lignin conformations from crumpled-globules to random-coil states, thereby benefiting to lignin removal. Because this effect was invariant to the temperature, it was proposed that H_2_O/THF system was effective to fractionate and solubilize lignin from lignocellulose biomass even at low temperatures (Smith et al., 2016a,b). Three models for the transform from globule lignin to coil lignin were proposed, those are, one-state model, two-state model and three-state model. The free energy Δ*G* for the transition was obtained from the fitted kinetic constants, in which the slope and intercept of Δ*G*-temperature function were taken as the Δ*S* and Δ*H* values, respectively. The results showed that the best fit among these three models was the two-state model. Due to the negative Δ*G*, the globule-to-coil transition was spontaneous at room temperature, which was also both enthalphically and entropically favorable (Smith et al., 2016a,b).

## Hemicellulose Fractionation and Solubilization and Further Conversion to Furfural in H_2_O/THF System

Hemicellulose with low degree of polymerization can be fractionated and further converted to furfural. The solubilization of hemicellulose/xylan and the further conversion of the resulted derivatives have also been conducted in H_2_O/THF co-solvent. Smith et al. (2018) demonstrated that both H_2_O/THF co-solvent and single water solvent acted as “good” solvents for hemicellulose/xylan conversion. In H_2_O/THF co-solvent, the temperature-phase behavior determined the constitution of xylan solvation shell. When H_2_O and THF were immiscible in the temperature range of 333–418 K, THF was left from the solvation shell of fractionated hemicellulose/xylan. In contrast, both H_2_O and THF were present in the solvation shell of fractionated hemicellulose/xylan when the temperature was below and above this temperature regime. This implied that the fractionated hemicellulose/xylan solubilization in single water medium was just similar to that in H_2_O/THF co-solvent in a temperature range of 333–418 K, but was greatly different outside this temperature range.

In addition to H_2_O/THF single phasic system, it is known that the addition of NaCl to H_2_O/THF co-solvent leads to the formation of biphasic NaCl-H_2_O/THF system. NaCl-H_2_O/THF biphasic system has been proved to show excellent efficiency for furfural formation from hemicellulose/xylan, where the further degradation of furfural produced in aqueous phase to the unwanted by-products like polymer and humins could be significantly inhibited, due to the immediate transfer of furfural from aqueous phase to organic phase. For instance, Xu et al. (2018b) examined the effect of organic solvents [methyl isobutyl ketone (MIBK), toluene, THF, MeTHF, and so on] on the production of furfural from xylose by the catalysis of CrPO_4_. It was indicated that the addition of organic solvents could obviously promoted xylose conversion when compared to single water medium (Table 3). The highest furfural yield was achieved in NaCl-H_2_O/THF system, attributing to the highest partition coefficient of THF for furfural among the selected solvent systems, which also efficiently eliminated furfural degradation in the aqueous phase. In addition, it was indicated that the H_2_O/THF ratio substantially influenced the furfural yield, and H_2_O/THF system with a 10/30 (mL/mL) ratio of H_2_O to THF gave much higher furfural yield (88%) than that with 10/20 (mL/mL) and 10/40 (mL/mL) of H_2_O/THF ratio. Yang et al. (2012b) also reported the benefits of NaCl-H_2_O/THF biphasic system, in which a 75 mol% of furfural yield from xylose conversion was obtained with AlCl_3_ · 6H_2_O as catalyst under microwave heating.

**Table 3:** Effects of reaction systems on the production of furfural from xylose/xylan.

**Entry**	**Feedstock**	**Solvent system**	**Catalyst**	***T* (°C)**	**Furfural yield (%)**	**References**
1	Xylose	H_2_O	CrPO_4_	160	18	Xu et al., 2018b
2	Xylose	NaCl-H_2_O/*n*-Butanol (10/30,v/v)	CrPO_4_	160	48	Xu et al., 2018b
3	Xylose	NaCl-H_2_O/2-Butanol(10/30,v/v)	CrPO_4_	160	19	Xu et al., 2018b
4	Xylose	NaCl-H_2_O/Toluene(10/30,v/v)	CrPO_4_	160	71	Xu et al., 2018b
5	Xylose	NaCl-H_2_O/MIBK(10/30,v/v)	CrPO_4_	160	86	Xu et al., 2018b
6	Xylose	NaCl-H_2_O/MeTHF(10/30,v/v)	CrPO_4_	160	82	Xu et al., 2018b
7	Xylose	NaCl-H_2_O/THF(10/30,v/v)	CrPO_4_	160	88	Xu et al., 2018b
8	Xylose	NaCl-H_2_O/THF(10/20,v/v)	CrPO_4_	160	69	Xu et al., 2018b
9	Xylose	NaCl-H_2_O/THF(10/40,v/v)	CrPO_4_	160	43	Xu et al., 2018b
10	Xylose	NaCl-H_2_O/THF(1/4,v/v)	AlCl_3_ · 6H_2_O	140	75	Yang et al., 2012b
11	Xylose	H_2_O/THF	CO_2_	160	53.3	Özbek et al., 2018
12	Hemicellulose hydrolysate	H_2_O/THF	CO_2_	170	39.6	Özbek et al., 2018
13	Brown seaweed	H_2_O/THF (5/95,v/v)	H_3_PW_12_O_40_	180	33.8	Park et al., 2016
14	Wheat straw	H_2_O/THF/MIBK(1/1/1, v/v)	CO_2_	180	43	Morais et al., 2016

Besides NaCl, other reagents have also been introduced into H_2_O/THF co-solvent, affording a biphasic H_2_O/THF system. For example, Özbek et al. (2018) studied the production of furfural in an H_2_O/THF system with the addition of high-pressure CO_2_. It was found that, in the presence of THF, CO_2_ generated biphasic H_2_O/THF system, because gaseous CO_2_ had low solubility in THF, and was also insoluble in water medium. Consequently, the formed furfural could be continuously transferred from aqueous phase to CO_2_/THF phase. Under the optimal pretreatment conditions, the highest furfural yield was up to 53.3 mol% when a model solution (including xylose and acetic acid) was used as starting material. In the case of real hemicellulose hydrolysate as feedstock, the yield of furfural was 39.6 mol% with 40.0 mol% of selectivity. Morais et al. (2016) proposed a two-stage strategy for the production of furfural from raw biomass. The first step involved the production of a water-soluble fraction (including xylose and xylo-oligosaccharide) via extracting hemicellulose component in wheat straw in H_2_O system with high-pressure CO_2_. In the second step, the resulted liquid fraction was further converted to furfural in H_2_O/THF/MIBK-high-pressure CO_2_ system, where MIBK was considered as the extracting solvent. However, it was found that the conversion of xylose decreased by 5 mol% in the presence of THF than that in a single water solvent. This was possibly due to the lower dielectric constant of H_2_O/THF (ε = 40) than that of pure water (ε = 78.5 at 25°C) which decreased the acidity of reactions system (Critchfield et al., 1953; Muinasmaa et al., 1997). In final, furfural with 56.6 mol% of yield and 62.3 mol% of selectivity was obtained in this H_2_O/THF/MIBK-high-pressure CO_2_ system (50 bar).

## Cellulose Fractionation and Solubilization in H_2_O/THF System

The presence of substantial hydrogen bonding in cellulose leads to a rigid structure with high crystallinity, which is strongly resistant to fractionation and dissolution and depolymerization in water. Several studies have demonstrated that polar aprotic solvents can simultaneously accelerate cellulose fractionation and dissolution and suppress the further dehydration reaction of monosaccharides, yielding target products with high yield and selectivity (Varhegyi et al., 1994; Ghosh et al., 2018). Typically, Ghosh et al. (2016) investigated the conversion of cellulose in a series of polar aprotic solvents, such as GVL, IMBK, acetonitrile, THF, 1,4-dioxane, ethyl acetate, and acetone. The results demonstrated that all the selected solvent fractionated and solubilized cellulose and gave desired products with high yield within a short time, possibly attributing to the decreased activation energy of cellulose depolymerization in polar aprotic solvents. The maximum yield of solubilized products from cellulose was 72–98% at 350°C. Combing the efficiency of solvents with their properties, it was revealed that the polar solubility parameter of a solvent might be the key factor contributing to its different fractionation and solubilization efficiency for cellulose (Archer, 1991; Su et al., 2009). Levoglucosan was the primary solubilized carbohydrate product, whose yield increased with increasing polar solubility parameter of solvents. The same group then investigated the efficiency of these polar aprotic solvents for the depolymerization of cellulose with the help of acid catalyst, producing water-soluble carbohydrates (Ghosh et al., 2018). The results indicated that acid catalyst significantly weakened the differences of yields in different solvents. In THF solvent, the levoglucosan selectivity at maximum yields reached 80%. Moreover, it was found that low rate of levoglucosan degradation was observed in those polar aprotic solvents with low polarity, thus enhancing the stability and promoting the yield of anhydrosugar.

**Figure 3:**
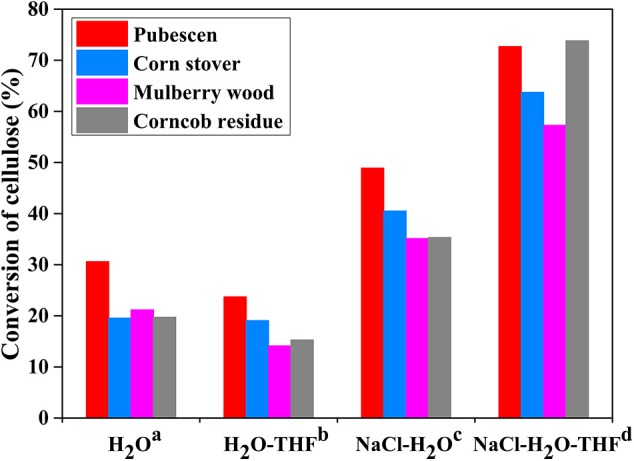
Conversion of cellulose in raw lignocellulose in different reaction systems. Reaction conditions: 4.0 g raw material and (a) 100 mL H_2_O; (b) 50 mL H_2_O and 50 mL THF; (c) 20 g NaCl and 100 mL H_2_O; (d) 20 g NaCl, 50 mL H_2_O and 50 mL THF; at 200°C for 2 h. Reproduced from Jiang et al. (2018) with permission from Wiley.

**Figure 4:**
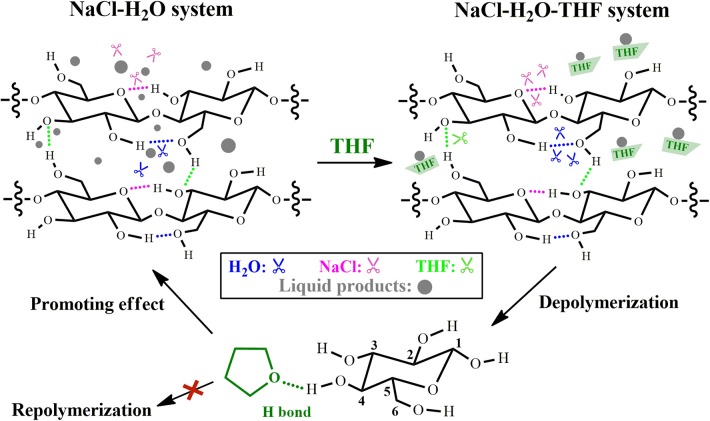
Effect of THF on the dissolution and depolymerization of M-cellulose in NaCl-H_2_O-THF system. Reaction conditions: M-cellulose (2.0 g) and NaCl (10 g) in solvent (100 mL, H_2_O–organic solvent: 1:1 v/v) at 200°C for 2 h. Reproduced from Jiang et al. (2018) with permission from Wiley.

Among the investigated polar aprotic solvent mixture with H_2_O, H_2_O/THF co-solvent system has been widely applied in the fractionation and solubilization/decrystallization of cellulose in lignocellulosic biomass (Zhang et al., 2014; Odabas et al., 2016). For instance, Jiang et al. (2016) pointed out that the introduction of THF into H_2_O could greatly enhance cellulose conversion from 17.2 to 45.9% with the assistance of heteropolyacid catalysts (ChH_2_PW_12_O_40_), which benefited the formation of HMF whose yield increased from 5.3 to 29.7%. Jiang et al. (2018) deeply investigated the performance of THF in NaCl-H_2_O/THF biphasic system via experimental approaches, and found that the conversion of microcrystalline cellulose (M-cellulose) could reach up to 96.6%, and the cellulose component in actual lignocelluloses could also be completely solubilized (Figure 3). In H_2_O/THF co-solvent system, it was revealed that THF could promote the cleavage of a half of intermolecular hydrogen bonds (O_6_-H···O_3_), while the role of H_2_O in breaking intra-molecular hydrogen bonds (O_2_-H···O_6_) was significantly impeded. In NaCl-H_2_O/THF biphasic system, THF could significantly improve the performances of both H_2_O and NaCl on the cleavage of O_2_-H···O_6_ and O_3_-H···O_5_ intra-molecular hydrogen bonds, respectively (Figure 4). Moreover, it was indicated that cellulose-derived products could be immediately transferred from aqueous phase to organic phase, ascribing to the formation of hydrogen bonds between O atom of THF and aldehyde group in HMF or H atom in C_4_-OH of glucose. This promoted the combination of more NaCl to –OH in M-cellulose, thus further disrupting the hydrogen bonding in M-cellulose and favoring the formation of products with small molecular weight (in particular HMF), which promoted the dissolution of cellulose in turn.

To elucidate the solubilization mechanism of cellulose in H_2_O/THF system, Mostofian et al. (2016) employed all-atom molecular dynamic simulation to investigate cellulose structure in H_2_O/THF mixture, as well as the interactions between cellulose and solvents. The results showed that H_2_O/THF co-solvent afforded different characteristics of both H_2_O and THF, resulting in the fractionation and solubilization of single cellulose chains and cellulose fibers via distinct ways. With the addition of THF to a water-only media, the reduction of cellulose hydration, especially for its hydrophobic faces, was observed, demonstrating that THF perturbed the interactions between H_2_O molecules and cellulose. In H_2_O/THF co-solvent system, H_2_O and THF spontaneously phase-separated on the surface of cellulose fiber, in which H_2_O accumulated at the hydrophilic cellulose faces due to the presence of hydrogen bonds between H_2_O molecules and cellulose on the hydrophilic faces of cellulose, while THF stacked on the hydrophobic faces of cellulose. In contrast, the cellulose chain with full solvation was preferentially bound by H_2_O molecules, indicating the easy hydrolysis of cellulose with the addition of THF. They also employed molecular dynamic simulations to compare the behavior of other H_2_O-organosolv co-solvent systems (acetone, ethanol, and GVL) with H_2_O/THF system (Smith et al., 2017). It was found that there were only weak differences in the selected solvent systems between the total amounts of hydrogen bonds in cellulose chain/strand-solvent and that of H_2_O molecules in the cellulose solvation shell. However, the selected H_2_O-organosolv co-solvents showed significantly distinct behavior of phase separation at the interface of co-solvent-cellulose, and the physical process of cellulose deconstruction was also different for these co-solvents. Particularly, in H_2_O/THF co-solvent, the fraction of surface water was much slower than that in H_2_O/GVL co-solvent, which might be important for promoting the chemical destroying of cellulose structure, since it could increase the reaction of THF-H_2_O-cellulose, even when H_2_O amount around cellulose was significantly decreased.

## Promoting Chemical Production from Lignocellulose in H_2_O/THF System

THF owns outstanding ability for the extraction of biomass-based chemicals, such as levulinic acid, HMF, and furfural, from water mixtures, thus significantly increasing the yield of target products and minimizing the generation of unwanted by-products like polymers and humins. The investigations of the catalytic conversion of lignocellulose to platform chemicals have shown that chemicals with high yield and selectivity could be achieved in H_2_O/THF system. For instance, Cai et al. (2013) reported THF could efficiently “protect” the formed furfural in aqueous phase from further degradation and minimizing furfural loss. In particular, NaCl-H_2_O/THF biphasic system significantly enhances the partitioning of target chemicals into organic phase, which has been successfully adopted to increase the yield of products derived from lignocellulosic feedstock (Xie et al., 2019). Saha and Abu-Omar (2014) reviewed the benefits of biphasic solvent system (including H_2_O/THF biphasic system) for the production of valuable chemicals (mainly HMF) from lignocellulose. In NaCl-H_2_O/THF biphasic solvent system, H_2_O is commonly recognized as the reactive phase for polysaccharide hydrolysis. The effects of THF can be classified into physical and chemical effects, wherein chemical effects involved the change of activation energy barriers in the key reaction step induced by the variant dynamics or environment in different solvent systems. The physical effect of THF related to the preferential solvation of the special functional groups in lignocellulosic derivatives, thus inhibiting their further conversion to form undesired by-products, in addition to immediate extracting the produced products from aqueous phase into organic phase once it was formed. Moreover, the extraction of chemicals in THF phase can improve the purity of obtained chemicals and make them be easily separated, thus enabling the treatment of lignocellulose economically and environmentally competitive. In this section, the roles of H_2_O/THF system in promoting the formation of several typical chemicals, including levoglucosan, HMF, levulinic acid, as well as the co-production of furfural and HMF, is attentively reviewed.

### Levoglucosan and Fermentable Sugar

Levoglucosenone (LGO), an anhydro-sugar with a double bond combined with a ketone and aldehyde, in addition to two hydroxyl groups (Tang et al., 2017b; Krishna et al., 2018), has shown a huge potential to serve as a bio-based platform chemical (Nguyen et al., 2015; He et al., 2017a; Li et al., 2018). Cao et al. (2015) found that the highest yields of LGO could be obtained in both THF and GVL aqueous co-solvents. However, LGO seemed to be more stable in THF because THF inhibited the further conversion of LGO to HMF. On the contrary, GVL favored the isomerization of LGO to HMF, leading to its degradation. In addition, it was demonstrated that the water content controlled the product distribution derived from cellulose, in which the increase of water content to 5 wt% led to HMF production, and no LGO was produced when using single water as solvent. They also compared the yield of LGO in H_2_O/THF with other H_2_O-organic co-solvents (such as diglyme, tetraglyme, cyclopentyl methyl ether, 1,4-dioxane, and dimethyl sulfoxide), and found that H_2_O/THF(1–2.5 wt% H_2_O) afforded the highest yields of HMF and LGO (~65 carbon%), with sulfuric acid as catalyst (He et al., 2017b). Weingarten et al. (2014) deeply investigated the influence of H_2_O/THF ratio on cellulose degradation with dilute sulfuric acid as catalyst, and indicated that higher carbon yield of LGO was obtained in both single THF solvent and H_2_O/THF co-solvent with a ratio of 40:1 (H_2_O/THF) compared to the other reaction mixtures. As for the TOF value of cellulose conversion, it was more than 20 times in THF higher than that in single water solvent. As for HMF generation in THF solvent, the TOF value was 40 times higher than that in single water medium. The promoting performance of THF under this reaction condition was attributed to the fact that proton catalyst was more stable in THF solvent than in water medium (by 5.7 kcal mol^−1^), thus resulting in the high reactivity of proton catalyst. Krishna et al. (2017) also showed that THF played a role in LGO stabilization and inhibited its isomerization reaction, thus resulting in the decrease of the yields of HMF and levulinic acid. The yield of xylose, arabinose, and glucose was up to 95% using corn stover as feedstock in H_2_O/THF aqueous dilute acid system.

### HMF Production

As for HMF production from lignocellulosic biomass, Weingarten et al. (2014) investigated the influence of a series of single organic solvents using H_2_SO_4_ as catalyst under mild conditions, and demonstrated that the polar aprotic solvents including THF, GVL, and acetone, gave a much higher HMF yield from cellulose when compared with water, ethyl acetate, and ethanol solvents. THF solvent afforded the highest HMF yield among the selected solvents. Compared to single THF solvent, the introduction of DMSO to THF solvent further improved the yield of HMF (98.0%). Wang et al. (2013) pointed out that the introduction of a small amount of water (<2.5 vol%) into THF was also beneficial for HMF production. The highest HMF yield from cellulose was 44%. In H_2_O/THF (1:4 v/v) co-solvent, the yields of fructose and HMF from glucose conversion were 61 and 30%, respectively, by the catalysis of a base (–NH_2_) functionalized mesoporous silica (aminopropyl-FMS) catalyst and a mesoporous silica with –SO_3_H (propylsulfonic acid-FMS) (Huang et al., 2014). Tucker et al. (2013) showed that the use of H_2_O/THF co-solvent resulted in the significant increase of HMF selectivity (>70%) with a 80% of fructose conversion.

Although HMF yield can be promoted in H_2_O/THF monophasic system, wherein the addition of a certain amount of H_2_O can favor the dissolution of polysaccharides in addition to promoting the degradation of LGO to produce HMF, HMF degradation to levulinic and formic acids would take place with further increasing H_2_O amount. To ensure the complete dissolution of feedstock in water but avoiding the degradation of HMF produced, NaCl–H_2_O/THF biphasic system appears to be a promising solution, which has been proved to efficiently inhibit the rehydration of HMF to levulinic acid in water by immediate extraction of HMF to organic phase (Table 4; Chen et al., 2016; Zhao et al., 2018a,b). Glucose is a typical feedstock for the production of HMF, with the help of various catalysts. For example, Nikolla et al. (2011) described the conversion of glucose to HMF using Sn-Beta with acid as catalysts in a H_2_O/THF biphasic system, and obtained the highest HMF selectivity (72%). Manganese phosphate (MnPO_4_) also exhibited good efficiency for HMF production from biomass-derived carbohydrates, and the yield of HMF was 59% using glucose as feedstock in H_2_O/THF biphasic reaction system (Xu et al., 2018a). Yang et al. (2012a) obtained a 61% yield of HMF from glucose by the catalysis of AlCl_3_ · 6H_2_O in NaCl-H_2_O/THF system at 160°C under microwave heating. They demonstrated that THF inhibited HMF rehydration to generate levulinic acid, and also decreased the yield of lactic acid. Moreover, THF also showed high partitioning coefficient for HMF, and HMF recovery reached up to 94% when using HMF as starting material. The same group next investigated the reaction kinetics of glucose conversion to HMF in water, H_2_O/THF, and NaCl–H_2_O/THF reaction solvents by the catalysis of AlCl_3_ (Tang et al., 2017a). Different product distributions were obtained in the three solvent systems, in which NaCl–H_2_O/THF biphasic system gave the highest yield and selectivity to HMF. The kinetics of possible reaction pathways in these three solvent systems was investigated. The results demonstrated that the rate constants in the kinetic model were very sensitive to the ratios of solvent compositions. In the H_2_O–THF co-solvent, the rates for fructose isomerization to glucose and formic acid formation from glucose and fructose were accelerated. On the contrary, the isomerization of glucose to fructose, fructose dehydration to HMF, and humins formation from fructose was decelerated. These results suggested that THF contributed to the inhibition of the rehydration and polymerization reactions of HMF, decreasing the formation of unwanted byproducts, as well as the dehydration of fructose to HMF and the polymerization of fructose to humins, but improved formic acid formation directly from glucose/fructose degradation without levulinic acid production. The yield of formic acid was therefore higher than that of levulinic acid in these selected solvents. Both in H_2_O/THF and NaCl–H_2_O/THF systems, the ratios of formic acid to levulinic acid were higher with lower levulinic acid yield, compared to that in water. Liang et al. (2019) indicated that HMF yield from glucose conversion in NaCl-H_2_O/THF biphasic system was higher than that in any single phase solvents by the catalysis of bifunctional porous polymer (PCP) [PCP(Cr)-NH_2−x_(CH_3_)_x_; *x* = 0, 1, or 2]. They also investigated the influence of H_2_O-to-THF ratio, and revealed that the yield of HMF greatly increased with the increase of H_2_O-to-THF ratio (from 1:1 to 1:2). In the case of 1:2 H_2_O-to-THF ratio, the yield of HMF was 65.9% with 99.9% of glucose conversion. However, the minor decrease of HMF yield was observed above this ratio, possibly ascribing to the reductive performance of catalyst with the addition of excessive THF. When using fructose as feedstock, fructose could transform to furaniod form, which could be converted to HMF more easily (Zhu et al., 2011). For instance, Yang et al. (2015) obtained the highest yield (71.5 mol%) of HMF when using fructose as starting material in biphasic H_2_O/THF system with FePO_4_ as catalyst.

**Table 4:** The production of HMF from carbohydrates in different reaction systems.

**Entry**	**Feedstock**	**Catalyst**	**Solvent system**	***T* (°C)**	**HMF yield (%)**	**References**
1	Glucose	THF	PCP(Cr)-NH_2−x_	180	4.5	Liang et al., 2019
2	Glucose	H_2_O	PCP(Cr)-NH_2−x_	160	30.7	Liang et al., 2019
3	Glucose	H_2_O	PCP(Cr)-NH_2−x_	180	36.6	Liang et al., 2019
4	Glucose	H_2_O	PCP(Cr)-NH_2−x_	200	30.2	Liang et al., 2019
5	Glucose	H_2_O/THF (1:2)	PCP(Cr)-NH_2−x_	180	58.3	Liang et al., 2019
6	Glucose	H_2_O/THF(1:1)	PCP(Cr)-NH_2−x_	190	56.4	Liang et al., 2019
7	Glucose	H_2_O/THF(1:2)	PCP(Cr)-NH_2−x_	190	65.9	Liang et al., 2019
8	Glucose	H_2_O/THF(1:3)	PCP(Cr)-NH_2−x_	190	64.3	Liang et al., 2019
9	Glucose	H_2_O/THF(1:4)	PCP(Cr)-NH_2−x_	190	52.0	Liang et al., 2019
10	Glucose	H_2_O/THF(1:2)	PCP(Cr)-NH_2−x_	200	60.7	Liang et al., 2019
11	Glucose	H_2_O	AlCl_3_·6H_2_O	160	22	Yang et al., 2012a
12	Glucose	NaCl-H_2_O/THF	AlCl_3_·6H_2_O	160	52	Yang et al., 2012a
13	Glucose	NaCl-H_2_O	AlCl_3_·6H_2_O	160	17	Yang et al., 2012a
14	Glucose	NaCl-H_2_O/THF	AlCl_3_·6H_2_O	160	61	Yang et al., 2012a
15	Glucose	NaCl-H_2_O/THF	HCl	160	12	Yang et al., 2012a
16	Glucose	H_2_O	Sn-Beta, HCl	160	2.7	Nikolla et al., 2011
17	Glucose	H_2_O/1-butanol	Sn-Beta, HCl	160	20.0	Nikolla et al., 2011
18	Glucose	NaCl-H_2_O/1-butanol	Sn-Beta	160	13.5	Nikolla et al., 2011
19	Glucose	NaCl-H_2_O/1-butanol	HCl	160	10.4	Nikolla et al., 2011
20	Glucose	NaCl-H_2_O/1-butanol	Sn-Beta, HCl	160	41.3	Nikolla et al., 2011
21	Glucose	NaCl-H_2_O/THF	Sn-Beta, HCl	180	56.9	Nikolla et al., 2011
22	Glucose	NaCl-H_2_O/THF	Ti-Beta, HCl	180	53.2	Nikolla et al., 2011
23	Glucose	H_2_O/THF	Aminopropyl-FMS, propylsulfonic acid-FMS	90	30	Huang et al., 2014
24	Glucose	NaCl-H_2_O/THF	MnPO_4_	160	59	Xu et al., 2018a
25	Fructose	NaCl-H_2_O/THF	FePO_4_	140	71.5	Yang et al., 2015
26	Cellobiose	NaCl-H_2_O/THF	Sn-Beta, HCl	180	13.0	Nikolla et al., 2011
27	Cellulose	THF	H_2_SO_4_	190	44	Yang et al., 2015
28	Cellulose	H_2_O/THF	NaHSO_4_/ZrO_2_	190	86.5	Fang et al., 2018
29	Bamboo fiber	H_2_O	–	180	Trace	Sun et al., 2015
30	Bamboo fiber	H_2_O	NH_2_SO_3_H	180	20.9	Sun et al., 2015
31	Bamboo fiber	H_2_O-NaCl	NH_2_SO_3_H	180	11.6	Sun et al., 2015
32	Bamboo fiber	H_2_O/THF(1:3)	NH_2_SO_3_H	180	30.7	Sun et al., 2015
33	Bamboo fiber	NaCl-H_2_O/THF(3:1)	NH_2_SO_3_H	180	11.3	Sun et al., 2015
34	Bamboo fiber	NaCl-H_2_O/THF(1:1)	NH_2_SO_3_H	180	39.7	Sun et al., 2015
35	Bamboo fiber	NaCl-H_2_O/THF(1:3)	NH_2_SO_3_H	180	52.2	Sun et al., 2015
36	Bamboo fiber	NaCl-H_2_O/THF(1:5)	NH_2_SO_3_H	180	49.2	Sun et al., 2015
37	Starch	NaCl-H_2_O/THF	Sn-Beta, HCl	180	51.8	Nikolla et al., 2011

In addition to glucose/fructose, the conversion of cellulose even raw lignocellulosic biomass could yield HMF in H_2_O/THF biphasic reaction system (Zhang et al., 2016; Yu et al., 2017). Fang et al. (2018) studied the production of HMF from cellulose in H_2_O/THF biphasic system with NaHSO_4_ and ZrO_2_. It was pointed out that the yield of HMF linearly increased when THF dosage was raised to 32 g, and kept stable with more THF addition. Instead, the yield of levulinic acid gave an opposite tendency to that of HMF with the increase of THF amount. The increasing THF amount also led to the enhancement of HMF extracting capacity. Consequently, the degradation of HMF to levulinic acid could be remarkably inhibited. More importantly, limited humins was formed with the addition of more THF, suggesting the efficient prevention of HMF condensation with glucose due to the transfer of HMF from aqueous phase to THF phase. The highest yield of HMF was up to 86.5%. Xuan et al. (2018) also showed that the volume ratio of H_2_O/THF could influence the conversion of M-cellulose to HMF in H_2_O/THF biphasic system with 1-(3-sulfonic acid)-propyl-3-methylimidazolium hydrogen sulfate ([PSMIM]HSO_4_) and ZnSO_4_·7H_2_O as catalyst, since altering the water content led to a change in catalyst concentration. The yields of HMF exhibited a volcano trend with increasing H_2_O volume, and a maximal value (58.8%) was achieved at a H_2_O/THF ratio of 2:20 (v/v), in which the side reactions in the aqueous phase was remarkably suppressed. Sun et al. (2015) investigated the production of HMF from lignocellulosic biomass by the catalysis of solid organic acid catalyst NH_2_SO_3_H (SA) in H_2_O/THF biphasic system with microwave heating, and found that THF contributed to suppress HMF rehydration, thus giving the highest HMF yield of 52.2%.

### The Co-production of HMF and Furfural

As we know, furfural is unstable and highly reactive, which can be further degraded especially in the presence of dilute acid at high temperature, therefore mild reaction conditions are generally benefited to reducing furfural degradation. On the contrary, the conversion rate of glucan/cellulose to HMF in pure water is much slower because of the slow rate of glucan hydrolysis, thus severer conditions are required to enhance the generation rate for HMF. When raw lignocellulose is employed as feedstock, the loss of furfural usually exceeds the rate of HMF generation. Therefore, the co-production of furfural and HMF is always challenging because the different requirement of activation barrier for HMF and furfural formation. Recently, it has been proved that H_2_O/THF co-solvent can overcome the practical barrier, which enables the simultaneous production of furfural and HMF from lignocellulose (Table 5). For example, Smith et al. (2018) observed that the formation of furfural was slower in H_2_O/THF system, although the rate of disappearance of xylose monomers was faster than that of bulk water. THF seemed to co-catalyze the dehydration reaction of both C5 and C6 sugars via a kinetically favorable pathway.

**Table 5:** The production of furfural and HMF from lignocellulosic feedstock^a^.

**Entry**	**Solvent**	**Substrate**	**Catalyst**	***T* (°C)**	**Yields (%)**		**References**
					**Furfural**	**HMF**	
1	None	Maple wood^b^	1 wt% H_2_SO_4_	170	62^a^	2.4^a^	Cai et al., 2013
2	1:3 THF-H_2_O	Maple wood^b^	1 wt% H_2_SO_4_	170	76^a^	4.9^a^	Cai et al., 2013
3	1:1 THF-H_2_O	Maple wood^b^	1 wt% H_2_SO_4_	170	87^a^	13^a^	Cai et al., 2013
4	3:1 THF-H_2_O	Maple wood^b^	1 wt% H_2_SO_4_	170	87^a^	21^a^	Cai et al., 2013
5	3:1 THF-H_2_O	Maple wood^b^	1 wt% H_2_SO_4_	170	86^a^	21^a^	Cai et al., 2013
6	None	Maple wood^b^	1 wt% H_2_SO_4_	170	39^a^	2.6^a^	Cai et al., 2013
7	1:1 THF-H_2_O	Maple wood^b^	1 wt% H_2_SO_4_	170	69^a^	7.6^a^	Cai et al., 2013
8	None	Maple wood^c^	1.5 wt% H_2_SO_4_	200	–	–	Cai et al., 2013
9	1:1 THF-H_2_O	Maple wood^b^	FeCl_3_	170	85^a^	16^a^	Cai et al., 2014a
10	1:1 THF-H_2_O	Maple wood^b^	CuCl_2_	170	83^a^	14^a^	Cai et al., 2014a
11	1:1 THF-H_2_O	Maple wood^b^	AlCl_3_	170	58^a^	18^a^	Cai et al., 2014a
12	1:1 THF-H_2_O	Maple wood^b^	CrCl_3_	170	43^a^	15^a^	Cai et al., 2014a
13	1:1 THF-H_2_O	Maple wood^b^	ZrOCl_2_	170	44^a^	14^a^	Cai et al., 2014a
14	1:1 THF-H_2_O	Corn stover^b^	H_2_SO_4_	170	84^a^	16^a^	Cai et al., 2014a
15	1:1 THF-H_2_O	Corn stover^b^	FeCl_3_	170	85^a^	12^a^	Cai et al., 2014a
16	1:1 THF-H_2_O	Corn stover^b^	ZrOCl_2_	170	38^a^	14^a^	Cai et al., 2014a
17	3:1 THF-H_2_O	Maple wood^b^	H_2_SO_4_	170	86^a^	21^a^	Cai et al., 2014a
18	3:1 THF-H_2_O	Maple wood^b^	FeCl_3_	170	97^a^	41^a^	Cai et al., 2014a
19	3:1 THF-H_2_O	Maple wood^b^	CuCl_2_	170	81^a^	22^a^	Cai et al., 2014a
20	3:1 THF-H_2_O	Maple wood^b^	AlCl_3_	170	75^a^	33^a^	Cai et al., 2014a
21	3:1 THF-H_2_O	Corn stover^b^	FeCl_3_	170	97^a^	42^a^	Cai et al., 2014a
22	3:1 THF-H_2_O	Corn stover^b^	CuCl_2_	170	89^a^	22^a^	Cai et al., 2014a
23	3:1 THF-H_2_O	Corn stover^b^	AlCl_3_	170	76^a^	36^a^	Cai et al., 2014a
24	4:1 THF-H_2_O	Maple wood^b^	FeCl_3_	170	95^a^	51^a^	Cai et al., 2014a
25	4:1 THF-H_2_O	Corn stover^b^	FeCl_3_	170	95^a^	45^a^	Cai et al., 2014a
26	NaCl-H_2_O/THF (1/3)	Wheat straw	SnCl_2_-PTA/β	180	71	30	Xu et al., 2019
27	NaCl-H_2_O/THF	Cellulose	AlCl_3_-HCl	180	92.2	44	Gomes et al., 2018
28	NaCl-H_2_O/THF	Cellulose	ZnCl_2_-HCl	180	81.4	36.5	Gomes et al., 2018
29	H_2_O/THF	Corn stover	H_2_SO_4_	190	81	76	Fang et al., 2019

a *The yield was based on the theoretical yield*.

b *5 wt% solid loading*.

c *10% total solid loading*.

Tan et al. (2018) investigated the production of furfural and HMF in different organic solvents, and found that THF media gave the highest yield (49.4%) of furfural, HMF and levulinic acid among the selected solvents over Hβ zeolite catalyst, followed by dioxane with the total yield of 43.8%. Due to the formation of char, both sulfolane and DMSO gave low yield of furfural, although the conversion of glucose was high. It was reported that Hβ zeolite catalyst showed a similar activity to a strong Brønsted acid, which depended on the solvation of proton relative to solvent polarity (Mellmer et al., 2014). Dioxane and THF solvents had smaller dipole moments, suggesting their weaker polarity compared with that of other solvents, thus resulting in less proton solvation and decreasing the degradation/polymerization of the generated HMF and furfural (Karinen et al., 2011; Hu et al., 2014). However, further increasing the water content in THF enhanced the rehydration of HMF forming levulinic acid and formic acid. Besides, the formation of by-products, such as polymer and humins resulted from the polymerization of sugars with HMF or levulinic acid could be also promoted owing to the increase of solution polarity. Cai et al. (2013) compared the yields of furfural, HMF, and levulinic acid derived from raw maple wood chips in H_2_O/THF co-solvent to that in non-solvent system, and indicated that THF promoted both the hydrolysis of polysaccharides in maple wood as well as the next dehydration reactions of C5 and C6 sugars. The control experiments by varying the H_2_O/THF ratio in H_2_O/THF co-solvent demonstrated that the highest yield was realized with 1:3 solutions, obtaining 86% furfural, 21% HMF, and 40% levulinic acid. They also achieved the highest total yields of HMF (51%) and furfural (95%) directly from lignocellulosic biomass using FeCl_3_ as catalyst in H_2_O/THF co-solvent, whereas the yield of levulinic acid was very low (6%) (Cai et al., 2014a).

In general, biphasic NaCl-H_2_O/THF system is more effective for the simultaneous production of furfural and HMF than miscible co-solvent, with the assistance of catalysts. In H_2_O/THF biphasic system, Fang et al. (2019) obtained 76 and 81% yields of HMF and furfural from corn stover, respectively, using H_2_SO_4_/Na_2_SO_4_ as catalyst. In combination of Lewis acid with Brønsted acids in NaCl-H_2_O/THF biphasic system, the yields of HMF and furfural was 44.0 and 92.2% catalyzed by AlCl_3_/HCl and 36.5 and 81.4% by the catalysis of ZnCl_2_/HCl, respectively (Gomes et al., 2018). As high as 71% yield of furfural and 30% yield of HMF could be simultaneously produced from wheat straw in NaCl-H_2_O/THF biphasic system catalyzed by SnCl_2_-PTA/β (Phosphotungstic acid) catalyst (Xu et al., 2019). Over NbOPO_4_ and Sn-Mont catalyst, the simultaneous production of furfural and HMF from lignocellulose in NaCl-H_2_O/THF biphasic system could be further converted to 2,5-dimethylfuran (DMF) and 2-methylfuran over Ru/Co_3_O_4_ catalyst, both of which could act as the promising liquid biofuels (Wang et al., 2014).

### Levulinic Acid

**Figure 5:**
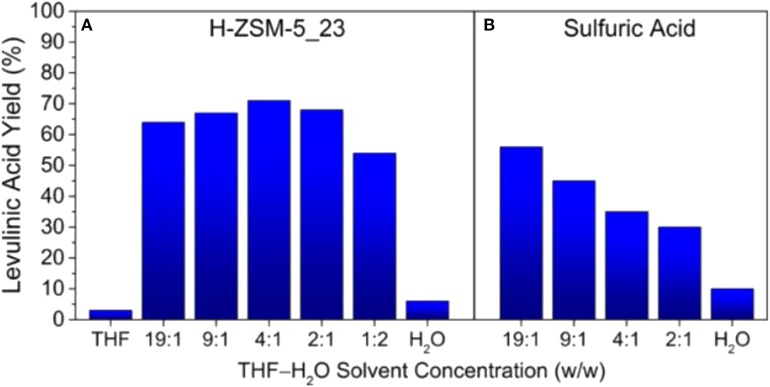
Maximum levulinic acid yields achieved at various H_2_O/THF solvent concentrations using **(A)** H-ZSM-5_23 and **(B)** sulfuric acid as catalyst. Reaction conditions: furfuryl alcohol (1 M), solvent (1.5 mL), 393 K, and stirring at 700 rpm. Reactant to solid catalyst ratio (w/w) = 0.6. Sulfuric acid solution = 0.1 M. Reproduced from Mellmer et al. (2015) with permission from American Chemistry Society.

The influence of different solvent systems, including water, THF and toluene solvents, on the production of levulinic acid from typical C6 sugar monomers/oligomers have also been investigated over solid acid catalysts, such as Amberlyst 70 (Hu et al., 2015). It was shown that solvent greatly influenced the yield of levulinic acid, in which the highest yield of levulinic acid was obtained in water, while the lowest yield was obtained in THF with dried A70 in toluene. This was possibly attributed to the different dispersion of sugars, products and catalyst in the solvents. In addition, solvent polarity affected the behaviors of Amberlyst 70, as well as the ability for the transfer of hydrogen ions and changing its dispersion in solvent. Mellmer et al. (2015) investigated the production of levulinic acid from furfuryl alcohol in a series of solvents, and indicated that the maximum levulinic acid yield (>70%) was achieved in monophasic H_2_O/THF (1:4, w/w) solvent system using HZSM-5 zeolite as catalyst (Figure 5). It was revealed that the hydrophobic feature of ZSM-5 could change the solvent microenvironment within the framework of zeolite, enabling the formation of levulinic acid with high yield, even though the concentration of THF was very low (2:1, H_2_O/THF, w/w). The reaction kinetic studies demonstrated that the increase of yield was attributed to the fact that the reaction rate could be significantly enhanced in polar aprotic solvents. The obtained levulinic acid could be further converted to GVL in THF medium. For example, 100% yield of GVL could be produced using molecular H_2_ over Ni/SA catalyst in THF medium, whereas much lower yield was found in water medium (Gundekari and Srinivasan, 2019).

## Inhibiting Humins Formation

**Figure 6:**
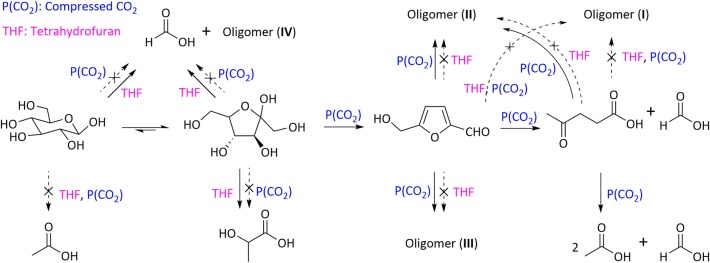
Role of compressed CO_2_ and THF in each subreaction of glucose conversion in a compressed CO_2_-H_2_O/THF solvent system. Reaction conditions: 2.2 mmol glucose, 32 mL solvent (H_2_O-THF 1:1), 1.0 MPa CO_2_, 190°C, 4 h. Reproduced from Fu et al. (2017) with permission from Royal Society of Chemistry.

One of the most important contributions of H_2_O/THF system for the improvement of both yield and selectivity to target products is relative to its role in inhibiting humins formation, which can also significantly enhance the carbon balance, as well as benefiting to the effective utilization of lignocellulose. This has been proved by substantial experimental results. For instance, Fu et al. (2017) showed that the presence of THF in CO_2_-H_2_O/THF system (1:1,V_H2O_/V_THF_) inhibited the generation of oligomers as well as increasing carbon balance. This suggested the promoting effect of THF on the retro-aldol condensation of fructose and the rehydration reaction of HMF, in addition to the inhibitory performance on the condensation of glucose via retro-aldol reaction (Figure 6). To deeply reveal the mechanism of H_2_O/THF co-solvent on the inhibition of humins formation, Vasudevan and Mushrif (2015) conducted molecular dynamics simulations to study glucose solvation in H_2_O/THF co-solvent. It was demonstrated that the added THF preferentially occupied the first solvation shell of glucose by competing with water molecules, in which most of water molecules were driven away from the first shell and existed in the second shell. Even in the presence of only a few THF, about 50% water molecules could be driven away from the first shell. Although the number of water molecules, which directly coordinated with glucose, became fewer with the addition of THF, the interaction between water molecules with glucose was indeed strengthened, where THF mainly localized around H atom of -OH in glucose. The preferential arrangement of water molecules and THF molecules around glucose might facilitate the conversion of glucose to HMF or levulinic acid, and also reduce the degradation of glucose to undesired by-products. Increasing the ratio of THF to H_2_O could also increase the lifetimes of hydrogen bond between glucose and water, thereby restricting the mobility of glucose in the solvent, thus reducing the formation rate of polymerization/condensation products and humins.

## Concluding Remarks and Perspective

In summary, H_2_O/THF co-solvent significantly fractionated and solubilized lignocellulosic biomass, especially for lignin and cellulose components, and lowered the recalcitrance of lignocellulose, affording liquid fluid with better mass and heat transfer, and enabling the adequate contact with catalyst, thereby favoring its further upgrading. In addition, H_2_O/THF system also promotes the hydrolysis of fractionated cellulose/hemicellulose to sugars, as well as the next sugar dehydration, thereby achieving high yields of valuable chemicals with high selectivity (e.g., furfural, HMF, and levulinic acid). More importantly, H_2_O/THF system, in particular NaCl-H_2_O/THF biphasic system, also own extraordinary properties for extracting these chemical compounds from water mixture, thus inhibiting their further conversion and significantly increasing the yield and selectivity of target products, and minimizing the formation of byproducts, such as polymerization products and humins. Therefore, H_2_O/THF system is a promising solvent system with multi-functions including not only the pretreatment and fractionation of lignocellulose but also the next conversion of solubilized oligomers to obtain valuable chemicals with high yield and selectivity. After reaction, THF could be separated and recycled by simple distillation, due to its lower boiling point, which could be reused for the next run. Thus, the valorization of biomass in H_2_O/THF system is considered as an economical approach.

There are still several problems which are not clarified and need deep investigation. For instance, the synergetic effect of H_2_O and organic solvent (e.g., THF) in the process of fractionated lignin or cellulose/hemicellulose solubilization, as well as the influence of solvent system on the structure and property of resulted liquid fluid which decides the yield and selectivity of downstream product, required further studies. In addition, the recent research has pointed out that co-solvent mixture with various organic solvent structure or different ratios of H_2_O to organic solvent exhibited unique selectivity for the solubilization of different components in lignocellulosic biomass. However, it is unknown about the reason and relationship between the properties of solvent system and its selectivity toward different components in lignocellulosic biomass. As for the further conversion of the resulted fluid, more information on the performance of solvent system needs to be revealed, besides the well-known extraction function. The influence of solvent system on the performance of catalyst also needs further clarification. In future work, we think more attention would be focused on the following aspects: (1) the influence of type and properties of solvent system on the selective fractionation and solubilization of one or two components in lignocellulosic biomass, (2) the deep elucidation of fractionation and solubilization in various solvents, (3) the performances of solvent system on the subsequent liquid fluid conversion, (4) mechanistic aspects at molecular level of the above conversion processes.

## Author Contributions

WZ and SX collaborated the references. JL and CH co-wrote and revised the paper.

### Conflict of Interest

The authors declare that the research was conducted in the absence of any commercial or financial relationships that could be construed as a potential conflict of interest.
